# Supplementary material for: Timing the initiation of sex: Delay mechanisms alter fitness outcomes in a rotifer population model
Source: J Theor Biol. Author manuscript; Available in PMC 2026 Jul 5. (PMC13333136; doi:10.1016/j.jtbi.2025.112333)
Supplement: 1 [file NIHMS2188354-supplement-1.pdf]

Supplementary Information for "Timing the initiation of sex: delay mechanisms alter fitness outcomes in a rotifer population model"

Bethany L. F. Stevens[a,*], Silke F. van Daalen[b], Tirzah J. Blomquist[c], Kristin E. Gribble[d], Michael G. Neubert[c]

[a]*Department of Ecology, Evolution, and Marine Biology. University of California Santa Barbara, Santa Barbara, CA, 93106, USA*
[b]*Wageningen Marine Research, 1976 CP, IJmuiden, NL*
[c]*Biology Department, Woods Hole Oceanographic Institution, Woods Hole, MA, 02543, USA*
[d]*Josephine Bay Paul Center for Comparative Molecular Biology and Evolution, Marine Biological Laboratory , Woods Hole, MA, 02543, USA*

Section S1

Figures S2-S6

*corresponding author: bstevens@ucsb.edu

S1. Distinguishing between two block implementations

We here present a simplified example to illustrate the difference between the effect of a temporal block on resting egg production and a generational block of the same duration, τ.

Consider a population of adults that may either be of the "stem" generation (those that initiate the population at time $t = 0$), $x_0(t)$, or of subsequent generations, $x_1(t)$. The total population of adults at time t is then, $x_0(t) + x_1(t)$.

All individuals in the population take τ days to mature to adulthood, are subject to mortality at the per capita rate q, and once mature, produce offspring at the rate 1 per time. If the initial size of the stem adult population is $x_0(0) = X_0$, then their population size thereafter decays exponentially:

$$x_0(t) = X_0 e^{-qt} \text{ for } t \geq 0. \tag{A.1}$$

Meanwhile, the population of adults of subsequent ("non-stem") generations changes according to

$$\frac{dx_1}{dt} = e^{-q\tau}\left[x_0(t-\tau) + x_1(t-\tau)\right] - qx_1(t), \text{ for } t \geq \tau, \tag{A.2}$$

with

$$x_1(t) = 0, \text{ for } t < \tau. \tag{A.3}$$

The first term on the right-hand side of equation (A.2) represents the maturation of individuals that survive to adulthood (born to stem or non-stem parents) and the second term describes the mortality of non-stem adults.

Now consider two scenarios in both of which this population generates resting eggs at a per capita rate $r > 0$. In the first scenario, resting eggs are produced only by non-stem adults. This is a generational block of length 1 generation. In this case, the cumulative number of eggs produced by the population up to time t will be

$$\tilde{y}(t) = \int_0^t rx_1(s)ds. \tag{A.4}$$

Because no non-stem individuals reach adulthood before time τ, we also have

$$\tilde{y}(t) = \int_\tau^t rx_1(s)ds. \tag{A.5}$$

In the second scenario, all adults in the population produce resting eggs after a temporal block equal in length to the maturation time τ. In this second case, the number of resting eggs produced will be

$$y(t) = \int_{\tau}^{t} r(x_1(s) + x_0(s))ds. \tag{A.6}$$

Substituting equation (A.1) for $x_0(s)$, equation (A.6) can be rewritten as

$$\begin{aligned} y(t) &= \int_{\tau}^{t} r(x_1(s) + X_0 e^{-qs})ds & \text{(A.7)} \\ &= \tilde{y}(t) + X_0 \frac{r}{q}(e^{-q\tau} - e^{-qt}). & \text{(A.8)} \end{aligned}$$

The second term in equation (A.8) is positive for all $t > \tau$, so $y(t) > \tilde{y}(t)$. That is, a temporal block produces more resting eggs than a comparable generational block for all t (Fig. S1A,B). In both scenarios, no resting eggs are produced until $t = \tau$, when either the length of the temporal block has passed or the first non-stem adults mature. Yet, the temporal block results in a faster rate of production once the block is lifted. The difference between $y(t)$ and $\tilde{y}(t)$ will vary with the initial size of the stem generation, X_0, the egg production rate, r, and the duration of the block, τ. If resting eggs form the initial population for subsequent growing seasons, egg production by a time-blocked population becomes exponentially larger than egg production by a generation-blocked population over consecutive seasons (Fig. S1C). These differences led us to construct a rotifer population growth model with a generational mictic block, the outputs of which we compare with the outputs of the temporal block model of Serra et al. (2005).

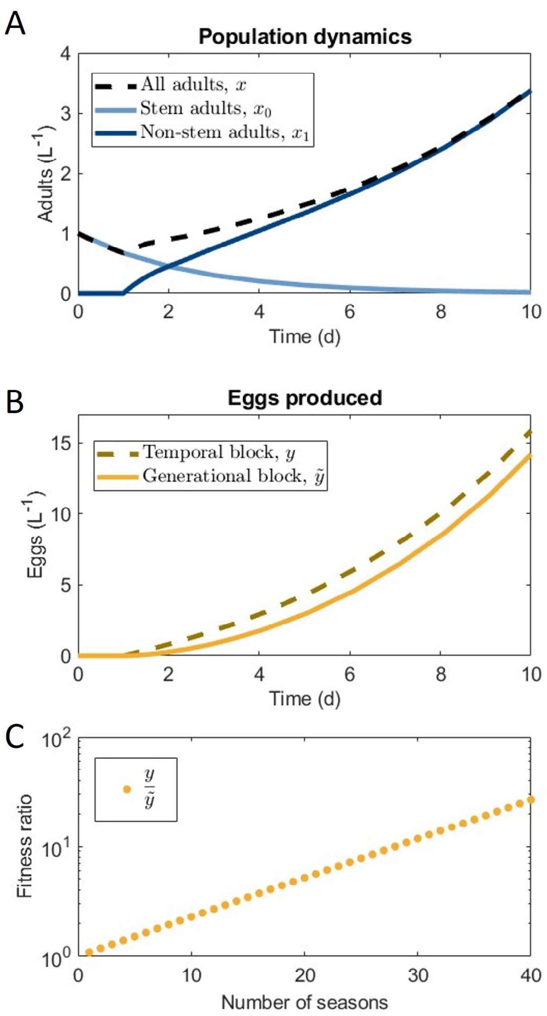

Figure S1: Difference in egg production between phenotypes with a temporal block and a generational block. (A) The number of adults in the population changes according to the model described in equations (A.1-A.3). (B) The number of eggs produced differs between the two different block implementations and depends on the initial size of the stem generation, the egg production rate, and the length of the block; however, the number of eggs produced in the temporal block scenario (dashed) is always greater than that in the generational block scenario (solid). (C) The fitness ratio between the two strategies increases exponentially with the number of growing seasons. Parameter values for these simulations are $X_0 = 1, \tau = 1$, and $q = 0.4$. Season length is 10 days.

Supplementary Figures

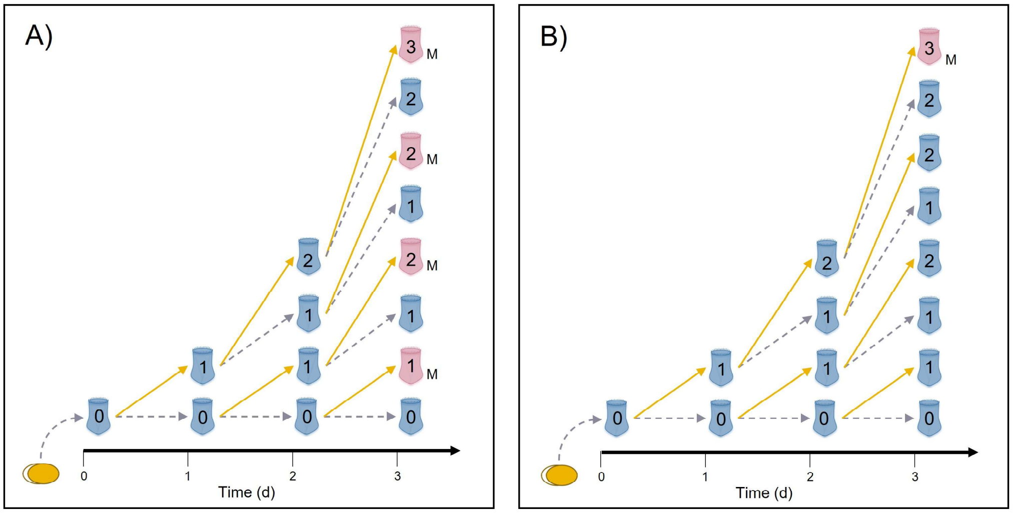

Figure S2: Example population where all rotifers produce one offspring each day demonstrating the difference between (A) a temporal block that lasts two days and (B) a generational block that lasts two generations. An initial resting egg hatches from diapause at $t = 0$. Gray dashed arrows indicate survival and yellow solid arrows indicate reproduction. The number overlaid on each individual indicates how many generations it is removed from the resting egg. For the sake of demonstration, we assume no maturation time, no mixis threshold density, and a mixis ratio of 1. In case (A) the block ends at $t = 2$ and all subsequently produced rotifers are mictic, indicated with an "M". In (B) only amictic individuals of generation 2 can produce mictic offspring. At day three, there are four mictic individuals in (A) and only one in (B).

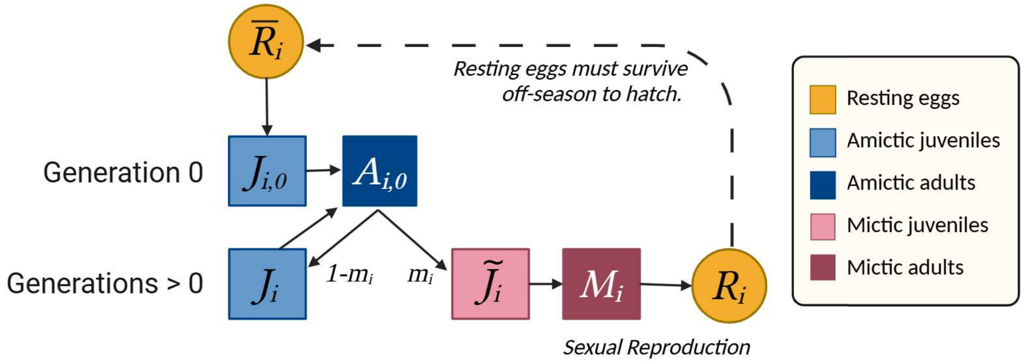

Figure S3: Version of model schematic in Fig., 2, but with $G_i = 0$. This is the case where the rotifer population has no mictic block. Note that there is still a mictic delay between resting egg hatching and new resting egg production that consists of the maturation time of stem and then mictic juveniles.

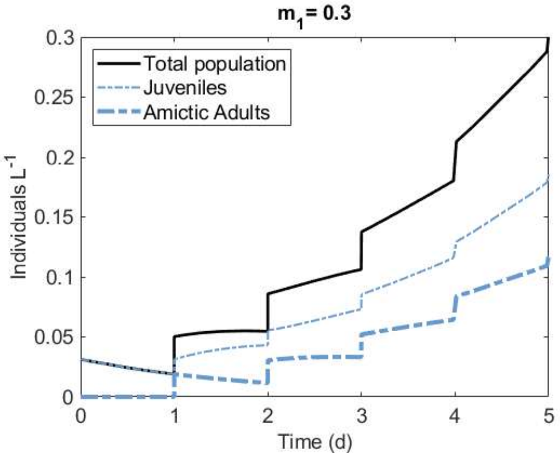

Figure S4: close-up of Fig. 3A highlighting the discontinuities in growth that result from hatching and maturing dynamics. At discrete intervals (in this case daily), new juveniles hatch and juveniles that have survived to adulthood mature.

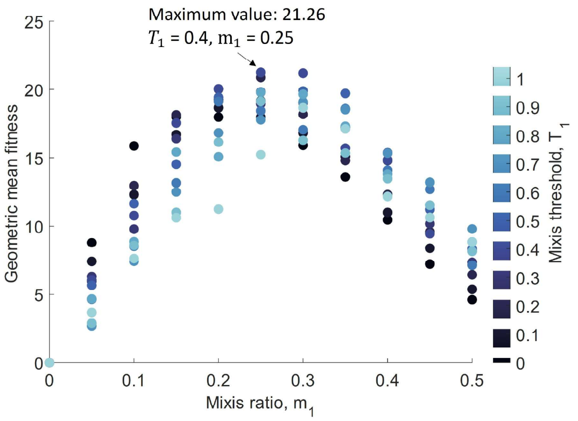

Figure S5: Fitness of monomorphic populations of phenotypes without a generational mictic block at a finer scale than Figure 4A. When $G_1 = 0$, the strategy with the highest average fitness has a mixis ratio of 0.25 at a resolution of 0.05 and a mixis threshold density of 0.4 at a resolution 0.1.

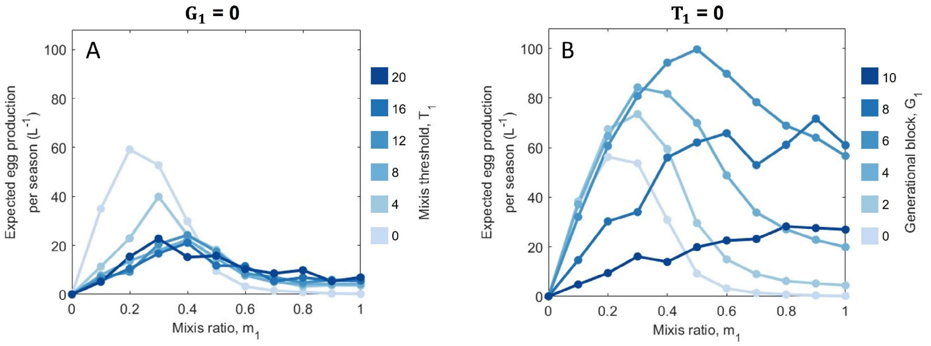

Figure S6: Expected egg production per season by monomorphic populations (A) without a generational mictic block and (B) without a mixis threshold density. Values plotted are the average eggs produced in 40 consecutive seasons of stochastic length (between 10 and 51 days) across 40 replicates with initial resting egg density equal to B_{max}. All parameters not indicated within the figure have values shown in Table 1.

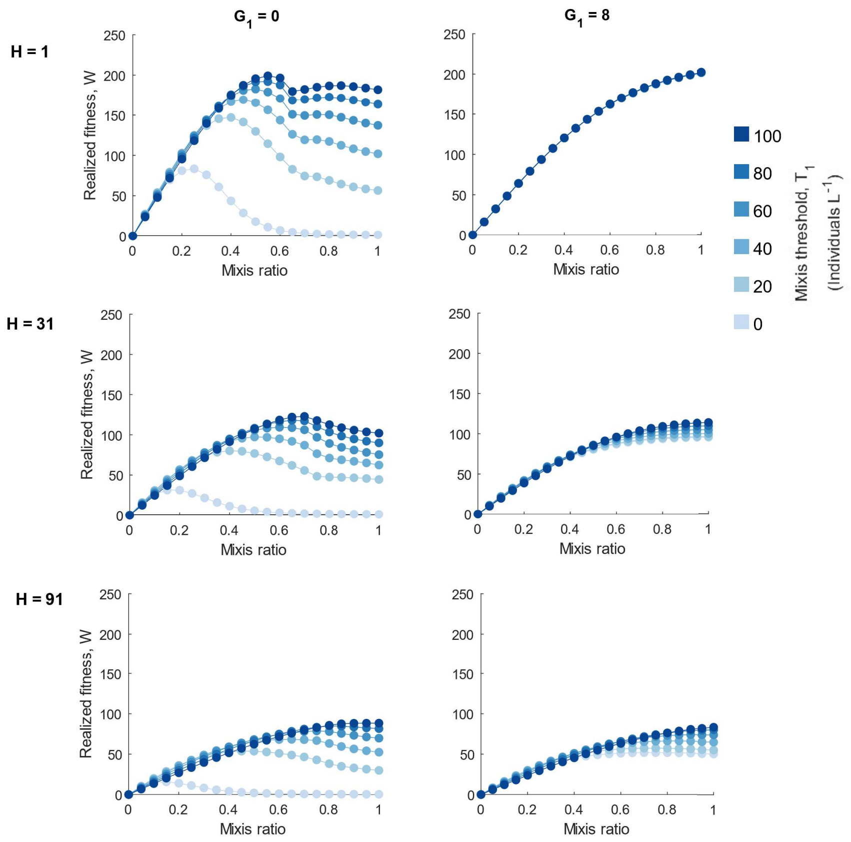

Figure S7: Realized fitness of monomorphic populations with multiple values of H (the end of the resting egg hatching period) indicated for each row. Seasons of length 30 days were simulated with all other parameter values indicated in Table 1 and initial resting egg density equal to $B_{max} = 1$. Populations that hatch earlier (low H), have similar fitness to standard populations in longer seasons (Fig. 5E), but trends across mixis phenotypes are unchanged.

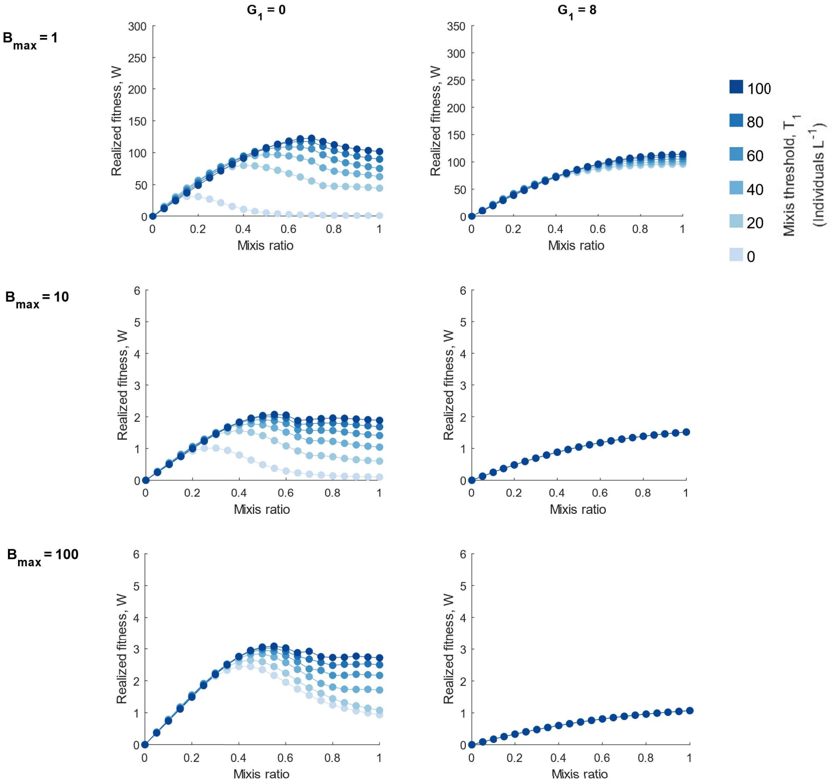

Figure S8: Realized fitness of monomorphic populations with multiple values of B_{max} (the initial size of the resting egg pool and maximum that can survive between seasons) indicated for each row. Seasons of length 30 days were simulated with all other parameter values indicated in Table 1.

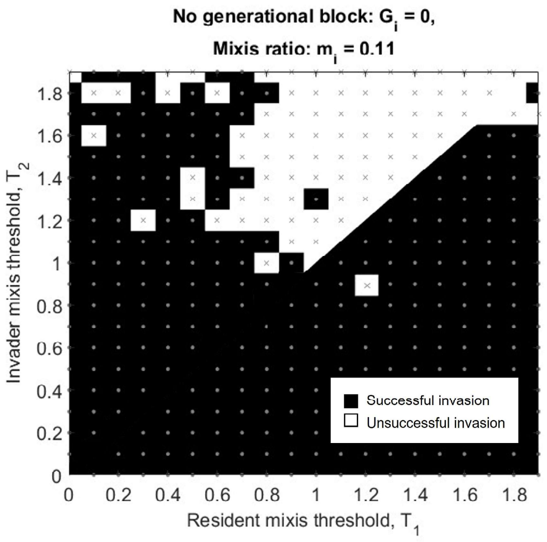

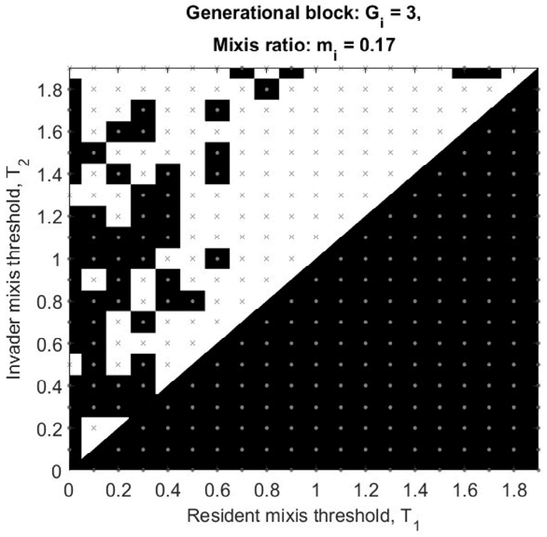

Figure S9: Pairwise invasibility plot for rotifer phenotypes with variable mixis threshold densities. As in Fig. 6, invasion was deemed successful if, out of 20 simulations of 40 growing seasons each, the invader frequency increased on average from its initial frequency of 0.05. White squares and cross marks indicate unsuccessful invasions, black sqaures and circle marks indicate successful invasion. Generally phenotypes with lower mixis threshold densities are more likely to successfully invade, and phenotypes with no mixis threshold density ($T_i = 0$) are able to invade all others. All parameters not indicated within the figure have values shown in Table 1

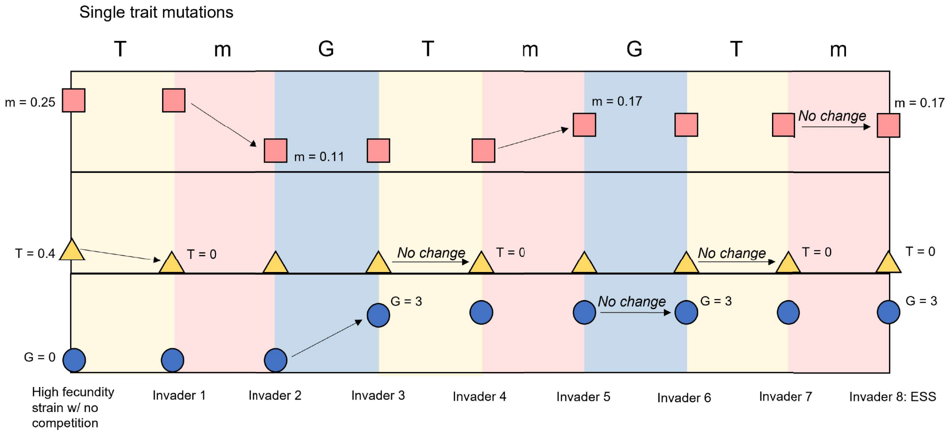

Figure S10: Schematic of sequential mutation simulation. We simulate a community evolving by allowing each dimension of the phenotype to change independently. For each single invasion step (columns), we construct a pairwise invasibility plot (Figs. S9,S11) and replace the resident with the invader that has a stable strategy along that single dimension. After five steps, the community reaches a phenotype that cannot be replaced by phenotypes with any single mutation.

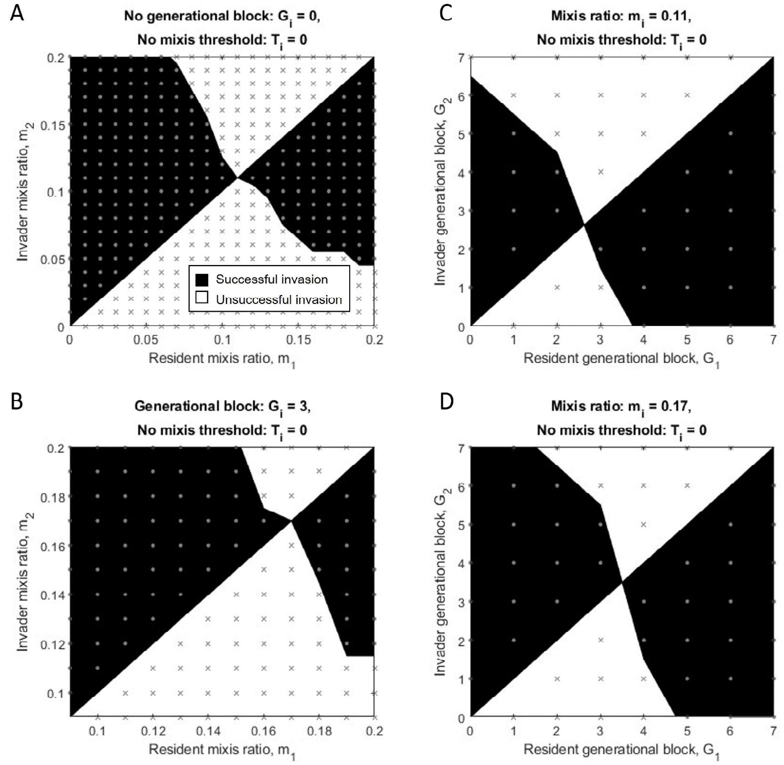

Figure S11: Pairwise invasibility plots for rotifers with A-B) variable mixis ratio and C-D) variable generational block. Panels A and C are exactly replicated from Fig. 6. As before, invasion was deemed successful if, out of 20 simulations of 40 growing seasons each, the invader frequency increased on average from its initial frequency of 0.05. Gray crosses indicate unsuccessful invasions; circles indicate successful invasions. Black and white regions are interpolated regions of success and failure, respectively. All parameters not indicated within the figure have values shown in Table 1.

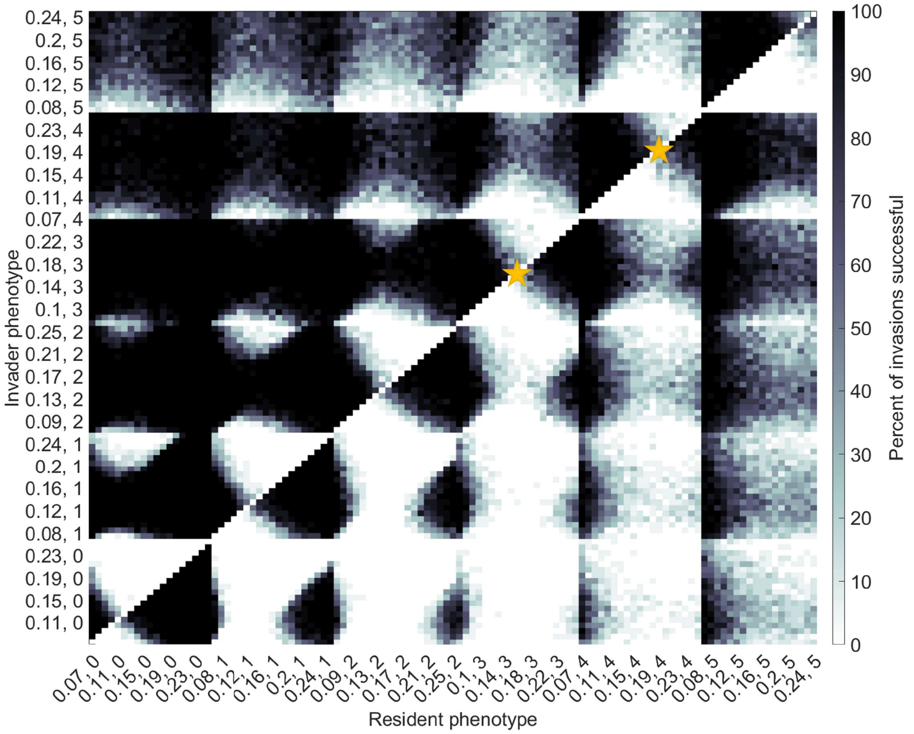

Figure S12: Pairwise invasibility plots for rotifers with variable mixis ratio and generational block length. Axes tick labels are values of m_i and G_i separated by a comma. Grid color shows percent of successful invasions out of 20 simulation experiments of 40 growing seasons each. Yellow stars indicate the two phenotypes that, on average, had highest invasion success and resistance to invasion by the other phenotypes within the entire grid shown.

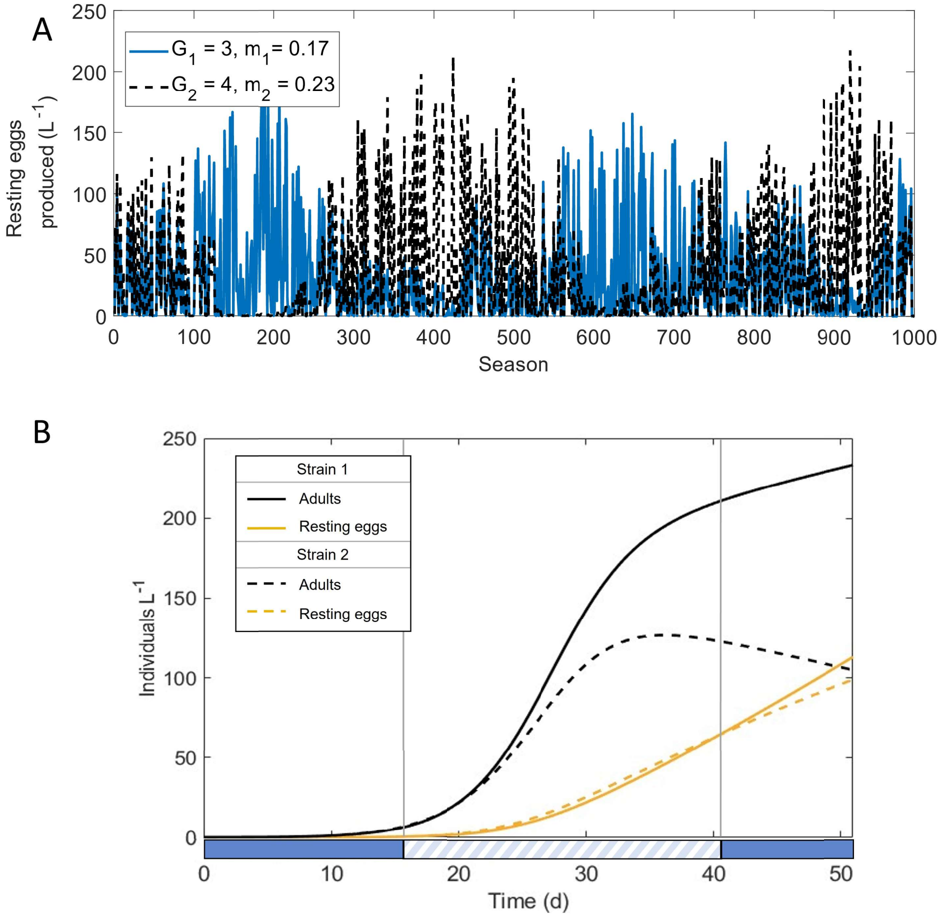

Figure S13: Population trajectories A) across seasons and B) within a single season for two phenotypes that can coexist within our standard environmental regime (uniformly distributed season lengths between 10 and 51 days). Solid lines represent the phenotype with shorter mictic block ($G_1 = 3$) and lower mixis ratio ($m_1 = 0.17$). Dashed lines represent a phenotype with longer mictic block ($G_2 = 4$) and higher mixis ratio ($m_2 = 0.23$). The abundance of the two phenotypes are equal ($\frac{1}{2}B_{max}$) at the start of the simulation. Vertical gray lines and shaded bars along the x-axis indicate the two points in time when the density of resting eggs for the two strategies intersect. Mixis threshold density, T_i, is 0 and all other parameter values in these simulations are those in Table 1.

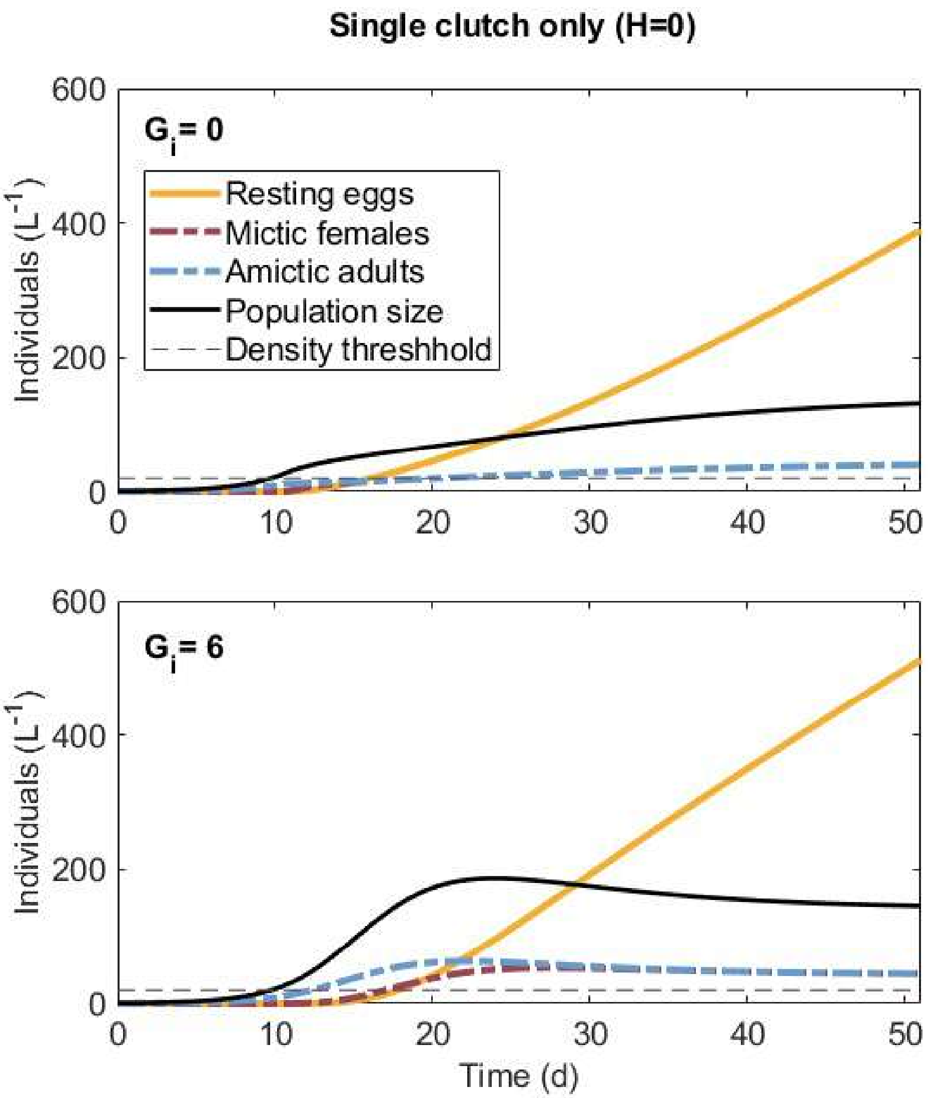

Figure S14: Population trajectories for monomorphic populations when all resting eggs hatch in a single clutch at t=0. Both phenotypes have a mixis ratio and threshold of 0.5 and 20 individual L^{-1}, respectively. Phenotypes with $G_i = 6$ (bottom) produce more resting eggs in a long season than those with no generational block (top). Benefits of generational block are not limited to the growth of late hatching clones.
